# Supplementary material for: Zooanthroponotic transmission of SARS-CoV-2 and host-specific viral mutations revealed by genome-wide phylogenetic analysis
Source: eLife. 2023 Apr 4;12:e83685. doi: 10.7554/eLife.83685 (PMC10072876; doi:10.7554/eLife.83685)
Supplement: Supplementary file 2. — The counts show the initial number of sequences downloaded from GISAID from each animal species, and the remaining number after each consecutive quality filter. The ‘quality control’ count shows the number of sequences after removing those with incomplete sampling dates and/or >500 ambiguous bases (Ns). The ‘post-alignment pruning’ shows the count after removing sequences shorter than 29,000 bases and/or with an insertion absent in all other sequences (introducing a gap in the alignment). The ‘divergent tree branches’ shows the count after removing sequences that introduce long branches into the phylogeny (Methods). Ranges of counts indicate variation across tree replicates. [file elife-83685-supp2.docx]

| *Species* | *Raw downloaded*  *(N sequences)* | *Post-quality control* | *Post-alignment pruning* | *Post-divergent tree branch pruning* |
| --- | --- | --- | --- | --- |
| mink | 1339 | 1046 | 1038 | 1036 |
| deer | 156 | 153 | 134 | 129-133 |
| cat | 120 | 100 | 78 | 78 |
| dog | 76 | 59 | 39 | 38-39 |
