# Supplementary material for: Zooanthroponotic transmission of SARS-CoV-2 and host-specific viral mutations revealed by genome-wide phylogenetic analysis
Source: eLife. 2023 Apr 4;12:e83685. doi: 10.7554/eLife.83685 (PMC10072876; doi:10.7554/eLife.83685)
Supplement: Supplementary file 3. [file elife-83685-supp3.docx]

**Table S3**. Table of transmission counts for all candidate species, in both animal-to-human and human-to-animal direction, for both bootstrap-filtered and unfiltered cases.

| **species** | **replicate** | **filtered count (animal to human)** | **unfiltered count (animal to human)** | **filtered count (human to animal)** | **unfiltered count (human to animal)** |
| --- | --- | --- | --- | --- | --- |
| **Cat** | 1 | 5 | 5 | 59 | 69 |
| **Cat** | 2 | 4 | 4 | 60 | 68 |
| **Cat** | 3 | 5 | 5 | 58 | 68 |
| **Cat** | 4 | 4 | 4 | 57 | 69 |
| **Cat** | 5 | 5 | 5 | 57 | 68 |
| **Cat** | 6 | 5 | 5 | 57 | 68 |
| **Cat** | 7 | 4 | 4 | 59 | 69 |
| **Cat** | 8 | 4 | 4 | 61 | 68 |
| **Cat** | 9 | 4 | 4 | 62 | 68 |
| **Cat** | 10 | 4 | 4 | 55 | 68 |
| **Dog** | 1 | 2 | 3 | 32 | 35 |
| **Dog** | 2 | 1 | 1 | 31 | 36 |
| **Dog** | 3 | 1 | 1 | 31 | 36 |
| **Dog** | 4 | 2 | 2 | 30 | 36 |
| **Dog** | 5 | 2 | 3 | 32 | 35 |
| **Dog** | 6 | 2 | 3 | 32 | 35 |
| **Dog** | 7 | 1 | 1 | 32 | 36 |
| **Dog** | 8 | 1 | 1 | 29 | 36 |
| **Dog** | 9 | 1 | 1 | 35 | 36 |
| **Dog** | 10 | 1 | 1 | 31 | 36 |
| **Mink** | 1 | 35 | 121 | 48 | 67 |
| **Mink** | 2 | 40 | 110 | 36 | 63 |
| **Mink** | 3 | 37 | 107 | 35 | 66 |
| **Mink** | 4 | 37 | 108 | 48 | 66 |
| **Mink** | 5 | 38 | 104 | 45 | 68 |
| **Mink** | 6 | 33 | 94 | 42 | 65 |
| **Mink** | 7 | 42 | 125 | 37 | 63 |
| **Mink** | 8 | 36 | 116 | 39 | 68 |
| **Mink** | 9 | 38 | 110 | 46 | 64 |
| **Mink** | 10 | 44 | 128 | 47 | 62 |
| **Deer** | 1 | 0 | 2 | 37 | 57 |
| **Deer** | 2 | 1 | 1 | 39 | 56 |
| **Deer** | 3 | 1 | 1 | 40 | 59 |
| **Deer** | 4 | 2 | 3 | 35 | 57 |
| **Deer** | 5 | 0 | 0 | 38 | 55 |
| **Deer** | 6 | 1 | 1 | 40 | 54 |
| **Deer** | 7 | 0 | 2 | 37 | 55 |
| **Deer** | 8 | 0 | 0 | 40 | 62 |
| **Deer** | 9 | 1 | 2 | 39 | 55 |
| **Deer** | 10 | 1 | 2 | 38 | 42 |
