# Supplementary material for: Zooanthroponotic transmission of SARS-CoV-2 and host-specific viral mutations revealed by genome-wide phylogenetic analysis
Source: eLife. 2023 Apr 4;12:e83685. doi: 10.7554/eLife.83685 (PMC10072876; doi:10.7554/eLife.83685)
Supplement: Supplementary file 4. — Odds ratio and the p-values are reported following a Fisher’s exact test. GWAS hits with OR <1 or not significantly different from 1 are highlighted in green. [file elife-83685-supp4.docx]

**Table S4.** Human-derived sequence counts bearing each of the significant GWAS hits identified in deer inside and outside regions where deer sequences containing each mutation are found. Odds ratio and the p-values are reported following a Fisher’s exact test. GWAS hits with OR < 1 or not significantly different from 1 are highlighted in green.

| **Position** | **Alternate allele count in deer regions** | **Wildtype allele count in deer regions** | **Alternate allele count outside deer regions** | **Wildtype allele count outside deer regions** | **Frequency of alternate allele in deer regions** | **Frequency of alternate allele outside deer regions** | **p_value** | **Conclusion** |
| --- | --- | --- | --- | --- | --- | --- | --- | --- |
| **7303** | 433 | 838982 | 2226 | 10804361 | 0.000516101656531368 | 0.0002 | 8.6862e-56 | ratio higher in deer regions |
| **9430** | 1812 | 946458 | 9313 | 10688419 | 0.0019145065074203 | 0.0009 | 1.2416e-172 | ratio higher in deer regions |
| **14960** | 0 | 268814 | 266 | 11376922 | 0 | 2.3381e-05 | 0.0035 | ratio lower in deer regions |
| **20259** | 21 | 446625 | 417 | 11198939 | 4.70193115029387e-05 | 3.7236e-05 | 0.3171 | ratio not significantly different |
| **28016** | 20 | 213258 | 176 | 11432548 | 9.37831171632483e-05 | 1.5395e-05 | 8.6845e-10 | ratio higher in deer regions |
| **12073** | 210 | 321652 | 7184 | 11316956 | 0.000652879509532041 | 0.0006 | 0.6703 | ratio not significantly different |
| **29679** | 180 | 289240 | 2231 | 11354351 | 0.000622320564237312 | 0.0002 | 2.80664e-37 | ratio higher in deer regions |
| **5184** | 7012 | 238828 | 126835 | 11273327 | 0.0293600415361683 | 0.0112 | 0 | ratio higher in deer regions |
| **29750** | 1442 | 577400 | 8856 | 11058304 | 0.00249740214755802 | 0.0008 | 2.2500e-267 | ratio higher in deer regions |
| **7318** | 131 | 272041 | 1440 | 11372390 | 0.000481545061222389 | 0.0001 | 9.5330e-35 | ratio higher in deer regions |
| **16466** | 316970 | 860090 | 864781 | 9604161 | 0.368531200223232 | 0.0900 | 0 | ratio higher in deer regions |
| **7267** | 181 | 206553 | 3597 | 11435671 | 0.000876288410238534 | 0.0003 | 2.1368e-31 | ratio higher in deer regions |
| **210** | 307476 | 869584 | 854780 | 9614162 | 0.353589762461131 | 0.0889 | 0 | ratio higher in deer regions |
| **6730** | 32 | 20464 | 10034 | 11615472 | 0.00156372165754496 | 0.0007 | 0.0018 | ratio higher in deer regions |
| **27752** | 306844 | 870216 | 842927 | 9626015 | 0.35260670913888 | 0.0876 | 0 | ratio higher in deer regions |
| **11152** | 196 | 301370 | 9246 | 11335190 | 0.000650363340743936 | 0.0008 | 0.0013 | ratio lower in deer regions |
| **5822** | 7 | 39079 | 3456 | 11603460 | 0.00017912433787968 | 0.0003 | 0.2361 | ratio not significantly different |
| **9711** | 26 | 391615 | 1331 | 11253030 | 6.63917367822989e-05 | 0.0001 | 0.0020 | ratio lower in deer regions |
| **9679** | 79 | 561506 | 673 | 11083744 | 0.000140693064722372 | 6.0719 | 2.1058e-10 | ratio higher in deer regions |
| **7029** | 7 | 157363 | 2276 | 11486356 | 4.44831377134396e-05 | 0.0002 | 4.4009e-07 | ratio lower in deer regions |
| **29738** | 17 | 20479 | 11278 | 11614228 | 0.000830118658137604 | 0.0001 | 0.6520 | ratio not significantly different |
| **26767** | 318642 | 858418 | 871184 | 9597758 | 0.371196782919277 | 0.0908 | 0 | ratio higher in deer regions |
| **203** | 726 | 72179 | 19565 | 11553532 | 0.0100583272142867 | 0.0017 | 6.16135e-297 | ratio higher in deer regions |
| **12820** | 10 | 20486 | 1255 | 11624251 | 0.00048813824074978 | 0.0001 | 0.0001 | ratio higher in deer regions |
| **4540** | 322 | 195956 | 6718 | 11443006 | 0.00164322603033334 | 0.0006 | 7.7575e-55 | ratio higher in deer regions |
| **29666** | 1940 | 513027 | 27323 | 11103712 | 0.00378147738812967 | 0.0025 | 3.2392e-66 | ratio higher in deer regions |
