# Supplementary material for: Zooanthroponotic transmission of SARS-CoV-2 and host-specific viral mutations revealed by genome-wide phylogenetic analysis
Source: eLife. 2023 Apr 4;12:e83685. doi: 10.7554/eLife.83685 (PMC10072876; doi:10.7554/eLife.83685)
Supplement: Supplementary file 5. — Odds ratio and the p-values are reported following a Fisher’s exact test. GWAS hits with OR <1 or not significantly different from 1 are highlighted in green. [file elife-83685-supp5.docx]

**Table S5.** Human-derived sequence counts bearing each of the significant GWAS hits identified in mink inside and outside regions where mink sequences containing each mutation are found. Odds ratio and the p-values are reported following a Fisher’s exact test. GWAS hits with OR < 1 or not significantly different from 1 are highlighted in green.

| **Position** | **Alternate allele count in mink regions** | **Wildtype allele count in mink regions** | **Alternate allele count outside mink regions** | **Wildtype allele count outside mink regions** | **Frequency of alternate allele in mink regions** | **Frequency of alternate allele outside mink regions** | **p_value** | **Conclusion** |
| --- | --- | --- | --- | --- | --- | --- | --- | --- |
| **26047** | 23 | 239184 | 279 | 11406516 | 9.6160e-05 | 2.44597035589132e-05 | 1.11877702252856e-07 | ratio higher in mink regions |
| **12795** | 34 | 152594 | 340 | 11493034 | 0.0002 | 2.95831370550196e-05 | 2.75263696173362e-18 | ratio higher in mink regions |
| **23064** | 67 | 296468 | 5363 | 11344104 | 0.0002 | 0.000472756596730777 | 1.43477105492256e-11 | ratio lower in mink regions |
