# Supplementary material for: Zooanthroponotic transmission of SARS-CoV-2 and host-specific viral mutations revealed by genome-wide phylogenetic analysis
Source: eLife. 2023 Apr 4;12:e83685. doi: 10.7554/eLife.83685 (PMC10072876; doi:10.7554/eLife.83685)
Supplement: Supplementary file 6. — The counts are summed across all branches and all 10 tree replicates. [file elife-83685-supp6.docx]

**Table S6.** Number of times Mink GWAS hits appear along human-to-mink transmission branches. The counts are summed across all branches and all 10 tree replicates.

| **species** | **Position** | **Number of times site is mutated on human-to-mink transition branches** | **Number of times the nucleotide substitution is identical to the GWAS hit** |
| --- | --- | --- | --- |
| **mink** | 26047 | 47 | 47 |
| **mink** | 12795 | 51 | 51 |
| **mink** | 23064 | 15 | 15 |
