# Supplementary material for: Zooanthroponotic transmission of SARS-CoV-2 and host-specific viral mutations revealed by genome-wide phylogenetic analysis
Source: eLife. 2023 Apr 4;12:e83685. doi: 10.7554/eLife.83685 (PMC10072876; doi:10.7554/eLife.83685)
Supplement: Supplementary file 7. — The counts are summed across all branches and all 10 tree replicates. Yellow-colored rows are mutations that never appear on a human-to-animal transmission branch over all deer replicates. The orange-colored row corresponds to a nucleotide position mutated along human-to-animal transmission branches, but the substitution was never identical to the animal-associated allele identified by GWAS at that position. [file elife-83685-supp7.docx]

**Table S7.** Number of times Deer GWAS hits appear along human-to-deer transmission branches. The counts are summed across all branches and all 10 tree replicates. Yellow colored rows are mutations that never appear on a human-to-animal transmission branch over all deer replicates. The orange colored row corresponds to a nucleotide position mutated along human-to-animal transmission branches, but the substitution was never identical to the animal-associated allele identified by GWAS at that position.

| **species** | **Position** | **Number of times site is mutated on human-to-mink transition branches** | **Number of times the nucleotide substitution is identical to the GWAS hit** |
| --- | --- | --- | --- |
| **deer** | 7303 | 147 | 138 |
| **deer** | 9430 | 133 | 133 |
| **deer** | 14960 | 10 | 10 |
| **deer** | 20259 | 47 | 47 |
| **deer** | 28016 | 30 | 30 |
| **deer** | 12073 | 21 | 21 |
| **deer** | 29679 | 40 | 40 |
| **deer** | 5184 | 6 | 6 |
| **deer** | 29750 | 40 | 20 |
| **deer** | 7318 | 36 | 27 |
| **deer** | 16466 | 0 | 0 |
| **deer** | 7267 | 39 | 30 |
| **deer** | 210 | 0 | 0 |
| **deer** | 6730 | 0 | 0 |
| **deer** | 27752 | 7 | 7 |
| **deer** | 11152 | 26 | 26 |
| **deer** | 5822 | 11 | 11 |
| **deer** | 9711 | 26 | 26 |
| **deer** | 9679 | 15 | 15 |
| **deer** | 7029 | 14 | 14 |
| **deer** | 29738 | 3 | 3 |
| **deer** | 26767 | 0 | 0 |
| **deer** | 203 | 10 | 10 |
| **deer** | 12820 | 10 | 0 |
| **deer** | 4540 | 10 | 10 |
| **deer** | 29666 | 40 | 40 |
