# Supplementary material for: Zooanthroponotic transmission of SARS-CoV-2 and host-specific viral mutations revealed by genome-wide phylogenetic analysis
Source: eLife. 2023 Apr 4;12:e83685. doi: 10.7554/eLife.83685 (PMC10072876; doi:10.7554/eLife.83685)
Supplement: Supplementary file 8. — Insertions and deletions are not considered. For each of the studies, mutations reported in the main text or main tables are included. For the (Pickering et al., 2022) study, substitutions found in deer in their study and also appear at least once in the database of previously reported deer sequences (listed in the paper’s Supplementary file 2) are included. [file elife-83685-supp8.docx]

**Table S8.** SARS-CoV-2 mutations previously associated with non-human animal species in the literature. Insertions and deletions are not considered. For each of the studies, mutations reported in the main text or main tables are included. For the Pickering et al. (2022) study, substitutions found in deer in their study and also appear at least once in the database of previously reported deer sequences (listed in the paper’s supplementary table S2) are included.

| **Mutation** | **Species** | **Study** |
| --- | --- | --- |
| **S_N501T** | **mink** | [**https://www.cell.com/cell-reports/pdf/S2211-1247(22)00060-2.pdf**](https://www.cell.com/cell-reports/pdf/S2211-1247(22)00060-2.pdf) |
| **S_Y453F** | **mink** | [**https://www.cell.com/cell-reports/pdf/S2211-1247(22)00060-2.pdf**](https://www.cell.com/cell-reports/pdf/S2211-1247(22)00060-2.pdf) |
| **S_F486L** | **mink** | [**https://www.cell.com/cell-reports/pdf/S2211-1247(22)00060-2.pdf**](https://www.cell.com/cell-reports/pdf/S2211-1247(22)00060-2.pdf) |
| **S_L452M** | **mink** | [**https://www.nature.com/articles/s41467-021-27096-9**](https://www.nature.com/articles/s41467-021-27096-9) |
| **S_Y453F** | **mink** | [**https://www.nature.com/articles/s41467-021-27096-9**](https://www.nature.com/articles/s41467-021-27096-9) |
| **S_F486L** | **mink** | [**https://www.nature.com/articles/s41467-021-27096-9**](https://www.nature.com/articles/s41467-021-27096-9) |
| **S_N501T** | **mink** | [**https://www.nature.com/articles/s41467-021-27096-9**](https://www.nature.com/articles/s41467-021-27096-9) |
| **NSP2_T85I** | **deer** | [**https://www.nature.com/articles/s41564-022-01268-9#Sec2**](https://www.nature.com/articles/s41564-022-01268-9#Sec2) |
| **NSP2_T528I** | **deer** | [**https://www.nature.com/articles/s41564-022-01268-9#Sec2**](https://www.nature.com/articles/s41564-022-01268-9#Sec2) |
| **NSP3_S1437F** | **deer** | [**https://www.nature.com/articles/s41564-022-01268-9#Sec2**](https://www.nature.com/articles/s41564-022-01268-9#Sec2) |
| **NSP4_H313Y** | **deer** | [**https://www.nature.com/articles/s41564-022-01268-9#Sec2**](https://www.nature.com/articles/s41564-022-01268-9#Sec2) |
| **NSP4_L353F** | **deer** | [**https://www.nature.com/articles/s41564-022-01268-9#Sec2**](https://www.nature.com/articles/s41564-022-01268-9#Sec2) |
| **NSP4_S386F** | **deer** | [**https://www.nature.com/articles/s41564-022-01268-9#Sec2**](https://www.nature.com/articles/s41564-022-01268-9#Sec2) |
| **NSP5_K90R** | **deer** | [**https://www.nature.com/articles/s41564-022-01268-9#Sec2**](https://www.nature.com/articles/s41564-022-01268-9#Sec2) |
| **NSP6_V149F** | **deer** | [**https://www.nature.com/articles/s41564-022-01268-9#Sec2**](https://www.nature.com/articles/s41564-022-01268-9#Sec2) |
| **NSP6_L260F** | **deer** | [**https://www.nature.com/articles/s41564-022-01268-9#Sec2**](https://www.nature.com/articles/s41564-022-01268-9#Sec2) |
| **NSP8_L169F** | **deer** | [**https://www.nature.com/articles/s41564-022-01268-9#Sec2**](https://www.nature.com/articles/s41564-022-01268-9#Sec2) |
| **NSP12_P323L** | **deer** | [**https://www.nature.com/articles/s41564-022-01268-9#Sec2**](https://www.nature.com/articles/s41564-022-01268-9#Sec2) |
| **NSP13_V348L** | **deer** | [**https://www.nature.com/articles/s41564-022-01268-9#Sec2**](https://www.nature.com/articles/s41564-022-01268-9#Sec2) |
| **NSP14_T113I** | **deer** | [**https://www.nature.com/articles/s41564-022-01268-9#Sec2**](https://www.nature.com/articles/s41564-022-01268-9#Sec2) |
| **S_T22I** | **deer** | [**https://www.nature.com/articles/s41564-022-01268-9#Sec2**](https://www.nature.com/articles/s41564-022-01268-9#Sec2) |
| **S_H49Y** | **deer** | [**https://www.nature.com/articles/s41564-022-01268-9#Sec2**](https://www.nature.com/articles/s41564-022-01268-9#Sec2) |
| **DEL** | **deer** | [**https://www.nature.com/articles/s41564-022-01268-9#Sec2**](https://www.nature.com/articles/s41564-022-01268-9#Sec2) |
| **S_Q613H** | **deer** | [**https://www.nature.com/articles/s41564-022-01268-9#Sec2**](https://www.nature.com/articles/s41564-022-01268-9#Sec2) |
| **S_D614G** | **deer** | [**https://www.nature.com/articles/s41564-022-01268-9#Sec2**](https://www.nature.com/articles/s41564-022-01268-9#Sec2) |
| **ORF3a_Q57H** | **deer** | [**https://www.nature.com/articles/s41564-022-01268-9#Sec2**](https://www.nature.com/articles/s41564-022-01268-9#Sec2) |
| **ORF3a_V256V** | **deer** | [**https://www.nature.com/articles/s41564-022-01268-9#Sec2**](https://www.nature.com/articles/s41564-022-01268-9#Sec2) |
| **N_T391I** | **deer** | [**https://www.nature.com/articles/s41564-022-01268-9#Sec2**](https://www.nature.com/articles/s41564-022-01268-9#Sec2) |
| **NSP9_G37E** | **mink** | [**https://www.nature.com/articles/s41467-022-30698-6**](https://www.nature.com/articles/s41467-022-30698-6) |
| **S_F486L** | **mink** | [**https://www.nature.com/articles/s41467-022-30698-6**](https://www.nature.com/articles/s41467-022-30698-6) |
| **S_N501T** | **mink** | [**https://www.nature.com/articles/s41467-022-30698-6**](https://www.nature.com/articles/s41467-022-30698-6) |
| **S_Y453F** | **mink** | [**https://www.nature.com/articles/s41467-022-30698-6**](https://www.nature.com/articles/s41467-022-30698-6) |
| **ORF3a_L219V** | **mink** | [**https://www.nature.com/articles/s41467-022-30698-6**](https://www.nature.com/articles/s41467-022-30698-6) |
| **NSP3a_L1035F** | **deer** | [**https://www.nature.com/articles/s41467-022-30698-6**](https://www.nature.com/articles/s41467-022-30698-6) |
| **NSP2_T170Q** | **mink** | [**https://peerj.com/articles/10609/**](https://peerj.com/articles/10609/) |
| **NSP2_A192V** | **mink** | [**https://peerj.com/articles/10609/**](https://peerj.com/articles/10609/) |
| **NSP2_H208Y** | **cat** | [**https://peerj.com/articles/10609/**](https://peerj.com/articles/10609/) |
| **NSP2_R218C** | **mink** | [**https://peerj.com/articles/10609/**](https://peerj.com/articles/10609/) |
| **NSP2_A225T** | **mink** | [**https://peerj.com/articles/10609/**](https://peerj.com/articles/10609/) |
| **NSP2_E563V** | **mink** | [**https://peerj.com/articles/10609/**](https://peerj.com/articles/10609/) |
| **NSP3_D135Y** | **cat** | [**https://peerj.com/articles/10609/**](https://peerj.com/articles/10609/) |
| **NSP3_V234I** | **cat** | [**https://peerj.com/articles/10609/**](https://peerj.com/articles/10609/) |
| **NSP3_P278L** | **mink** | [**https://peerj.com/articles/10609/**](https://peerj.com/articles/10609/) |
| **NSP3_H295Y** | **mink** | [**https://peerj.com/articles/10609/**](https://peerj.com/articles/10609/) |
| **NSP3_K384N** | **cat** | [**https://peerj.com/articles/10609/**](https://peerj.com/articles/10609/) |
| **NSP3_I750V** | **mink** | [**https://peerj.com/articles/10609/**](https://peerj.com/articles/10609/) |
| **NSP3_M770K** | **mink** | [**https://peerj.com/articles/10609/**](https://peerj.com/articles/10609/) |
| **NSP3_D1283G** | **dog** | [**https://peerj.com/articles/10609/**](https://peerj.com/articles/10609/) |
| **NSP5_I249T** | **cat** | [**https://peerj.com/articles/10609/**](https://peerj.com/articles/10609/) |
| **NSP5_I259V** | **mink** | [**https://peerj.com/articles/10609/**](https://peerj.com/articles/10609/) |
| **NSP9_G37E** | **mink** | [**https://peerj.com/articles/10609/**](https://peerj.com/articles/10609/) |
| **NSP9_G37R** | **mink** | [**https://peerj.com/articles/10609/**](https://peerj.com/articles/10609/) |
| **NSP10_K124E** | **cat** | [**https://peerj.com/articles/10609/**](https://peerj.com/articles/10609/) |
| **NSP12_T25I** | **cat** | [**https://peerj.com/articles/10609/**](https://peerj.com/articles/10609/) |
| **NSP12_M195I** | **mink** | [**https://peerj.com/articles/10609/**](https://peerj.com/articles/10609/) |
| **NSP12_T802I** | **mink** | [**https://peerj.com/articles/10609/**](https://peerj.com/articles/10609/) |
| **NSP13_I258V** | **mink** | [**https://peerj.com/articles/10609/**](https://peerj.com/articles/10609/) |
| **NSP13_A446D** | **mink** | [**https://peerj.com/articles/10609/**](https://peerj.com/articles/10609/) |
| **NSP15_A92T** | **mink** | [**https://peerj.com/articles/10609/**](https://peerj.com/articles/10609/) |
| **S_G261D** | **mink** | [**https://peerj.com/articles/10609/**](https://peerj.com/articles/10609/) |
| **S_G261D** | **mink** | [**https://peerj.com/articles/10609/**](https://peerj.com/articles/10609/) |
| **S_A262S** | **mink** | [**https://peerj.com/articles/10609/**](https://peerj.com/articles/10609/) |
| **S_V367F** | **mink** | [**https://peerj.com/articles/10609/**](https://peerj.com/articles/10609/) |
| **S_L452M** | **mink** | [**https://peerj.com/articles/10609/**](https://peerj.com/articles/10609/) |
| **S_Y453F** | **mink** | [**https://peerj.com/articles/10609/**](https://peerj.com/articles/10609/) |
| **S_F486L** | **mink** | [**https://peerj.com/articles/10609/**](https://peerj.com/articles/10609/) |
| **S_N501T** | **mink** | [**https://peerj.com/articles/10609/**](https://peerj.com/articles/10609/) |
| **M_T175M** | **cat** | [**https://peerj.com/articles/10609/**](https://peerj.com/articles/10609/) |
| **N_R41L** | **mink** | [**https://peerj.com/articles/10609/**](https://peerj.com/articles/10609/) |
| **N_P80L** | **mink** | [**https://peerj.com/articles/10609/**](https://peerj.com/articles/10609/) |
| **N_P199Q** | **mink** | [**https://peerj.com/articles/10609/**](https://peerj.com/articles/10609/) |
| **N_T247I** | **cat** | [**https://peerj.com/articles/10609/**](https://peerj.com/articles/10609/) |
| **ORF3a_H182L** | **mink** | [**https://peerj.com/articles/10609/**](https://peerj.com/articles/10609/) |
| **ORF3a_H182Y** | **mink** | [**https://peerj.com/articles/10609/**](https://peerj.com/articles/10609/) |
| **ORF3a_L219V** | **mink** | [**https://peerj.com/articles/10609/**](https://peerj.com/articles/10609/) |
| **ORF3a_G224C** | **mink** | [**https://peerj.com/articles/10609/**](https://peerj.com/articles/10609/) |
| **ORF3a_T229I** | **mink** | [**https://peerj.com/articles/10609/**](https://peerj.com/articles/10609/) |
| **ORF6_R20S** | **mink** | [**https://peerj.com/articles/10609/**](https://peerj.com/articles/10609/) |
| **ORF6_K23S** | **mink** | [**https://peerj.com/articles/10609/**](https://peerj.com/articles/10609/) |
| **NSP2_T85I** | **mink** | [**https://journals.plos.org/plospathogens/article/figure?id=10.1371/journal.ppat.1009952.t001**](https://journals.plos.org/plospathogens/article/figure?id=10.1371/journal.ppat.1009952.t001) |
| **NSP3_S1206L** | **mink** | [**https://journals.plos.org/plospathogens/article/figure?id=10.1371/journal.ppat.1009952.t001**](https://journals.plos.org/plospathogens/article/figure?id=10.1371/journal.ppat.1009952.t001) |
| **NSP9_G37E** | **mink** | [**https://journals.plos.org/plospathogens/article/figure?id=10.1371/journal.ppat.1009952.t001**](https://journals.plos.org/plospathogens/article/figure?id=10.1371/journal.ppat.1009952.t001) |
| **NSP12_P323L** | **mink** | [**https://journals.plos.org/plospathogens/article/figure?id=10.1371/journal.ppat.1009952.t001**](https://journals.plos.org/plospathogens/article/figure?id=10.1371/journal.ppat.1009952.t001) |
| **NSP13_V187I** | **mink** | [**https://journals.plos.org/plospathogens/article/figure?id=10.1371/journal.ppat.1009952.t001**](https://journals.plos.org/plospathogens/article/figure?id=10.1371/journal.ppat.1009952.t001) |
| **NSP16_T91M** | **mink** | [**https://journals.plos.org/plospathogens/article/figure?id=10.1371/journal.ppat.1009952.t001**](https://journals.plos.org/plospathogens/article/figure?id=10.1371/journal.ppat.1009952.t001) |
| **S_N501T** | **mink** | [**https://journals.plos.org/plospathogens/article/figure?id=10.1371/journal.ppat.1009952.t001**](https://journals.plos.org/plospathogens/article/figure?id=10.1371/journal.ppat.1009952.t001) |
| **S_D614G** | **mink** | [**https://journals.plos.org/plospathogens/article/figure?id=10.1371/journal.ppat.1009952.t001**](https://journals.plos.org/plospathogens/article/figure?id=10.1371/journal.ppat.1009952.t001) |
| **S_K113T** | **mink** | [**https://journals.plos.org/plospathogens/article/figure?id=10.1371/journal.ppat.1009952.t001**](https://journals.plos.org/plospathogens/article/figure?id=10.1371/journal.ppat.1009952.t001) |
| **ORF3a_H128Y** | **mink** | [**https://journals.plos.org/plospathogens/article/figure?id=10.1371/journal.ppat.1009952.t001**](https://journals.plos.org/plospathogens/article/figure?id=10.1371/journal.ppat.1009952.t001) |
| **ORF3a_Q57H** | **mink** | [**https://journals.plos.org/plospathogens/article/figure?id=10.1371/journal.ppat.1009952.t001**](https://journals.plos.org/plospathogens/article/figure?id=10.1371/journal.ppat.1009952.t001) |
| **M_A38S** | **mink** | [**https://journals.plos.org/plospathogens/article/figure?id=10.1371/journal.ppat.1009952.t001**](https://journals.plos.org/plospathogens/article/figure?id=10.1371/journal.ppat.1009952.t001) |
| **N_T205I** | **mink** | [**https://journals.plos.org/plospathogens/article/figure?id=10.1371/journal.ppat.1009952.t001**](https://journals.plos.org/plospathogens/article/figure?id=10.1371/journal.ppat.1009952.t001) |
| **N_Q289H** | **mink** | [**https://journals.plos.org/plospathogens/article/figure?id=10.1371/journal.ppat.1009952.t001**](https://journals.plos.org/plospathogens/article/figure?id=10.1371/journal.ppat.1009952.t001) |
| **NSP6_L260F** | **mink** | [**https://insight.jci.org/articles/view/159573**](https://insight.jci.org/articles/view/159573) |
| **ORF1b_P314L** | **mink** | [**https://www.frontiersin.org/articles/10.3389/fmicb.2021.698944/full**](https://www.frontiersin.org/articles/10.3389/fmicb.2021.698944/full) |
| **ORF1b_T730I** | **mink** | [**https://www.frontiersin.org/articles/10.3389/fmicb.2021.698944/full**](https://www.frontiersin.org/articles/10.3389/fmicb.2021.698944/full) |
| **ORF1b_T2163I** | **mink** | [**https://www.frontiersin.org/articles/10.3389/fmicb.2021.698944/full**](https://www.frontiersin.org/articles/10.3389/fmicb.2021.698944/full) |
| **S_Y453F** | **mink** | [**https://www.frontiersin.org/articles/10.3389/fmicb.2021.698944/full**](https://www.frontiersin.org/articles/10.3389/fmicb.2021.698944/full) |
| **S_D614G** | **mink** | [**https://www.frontiersin.org/articles/10.3389/fmicb.2021.698944/full**](https://www.frontiersin.org/articles/10.3389/fmicb.2021.698944/full) |
| **S_I692V** | **mink** | [**https://www.frontiersin.org/articles/10.3389/fmicb.2021.698944/full**](https://www.frontiersin.org/articles/10.3389/fmicb.2021.698944/full) |
| **S_M1229I** | **mink** | [**https://www.frontiersin.org/articles/10.3389/fmicb.2021.698944/full**](https://www.frontiersin.org/articles/10.3389/fmicb.2021.698944/full) |
| **ORF3a_H128Y** | **mink** | [**https://www.frontiersin.org/articles/10.3389/fmicb.2021.698944/full**](https://www.frontiersin.org/articles/10.3389/fmicb.2021.698944/full) |
| **N_S194L** | **mink** | [**https://www.frontiersin.org/articles/10.3389/fmicb.2021.698944/full**](https://www.frontiersin.org/articles/10.3389/fmicb.2021.698944/full) |
| **N_R203K** | **mink** | [**https://www.frontiersin.org/articles/10.3389/fmicb.2021.698944/full**](https://www.frontiersin.org/articles/10.3389/fmicb.2021.698944/full) |
| **N_G204R** | **mink** | [**https://www.frontiersin.org/articles/10.3389/fmicb.2021.698944/full**](https://www.frontiersin.org/articles/10.3389/fmicb.2021.698944/full) |
