# Supplementary material for: Zooanthroponotic transmission of SARS-CoV-2 and host-specific viral mutations revealed by genome-wide phylogenetic analysis
Source: eLife. 2023 Apr 4;12:e83685. doi: 10.7554/eLife.83685 (PMC10072876; doi:10.7554/eLife.83685)
Supplement: Figure 2—source data 1. — In order to make the tree topology clear, branch lengths are not to scale. [file eLifeFigure2Figure Supplement 1.pdf › eLifeFigure2Figure Supplement 1.pdf]

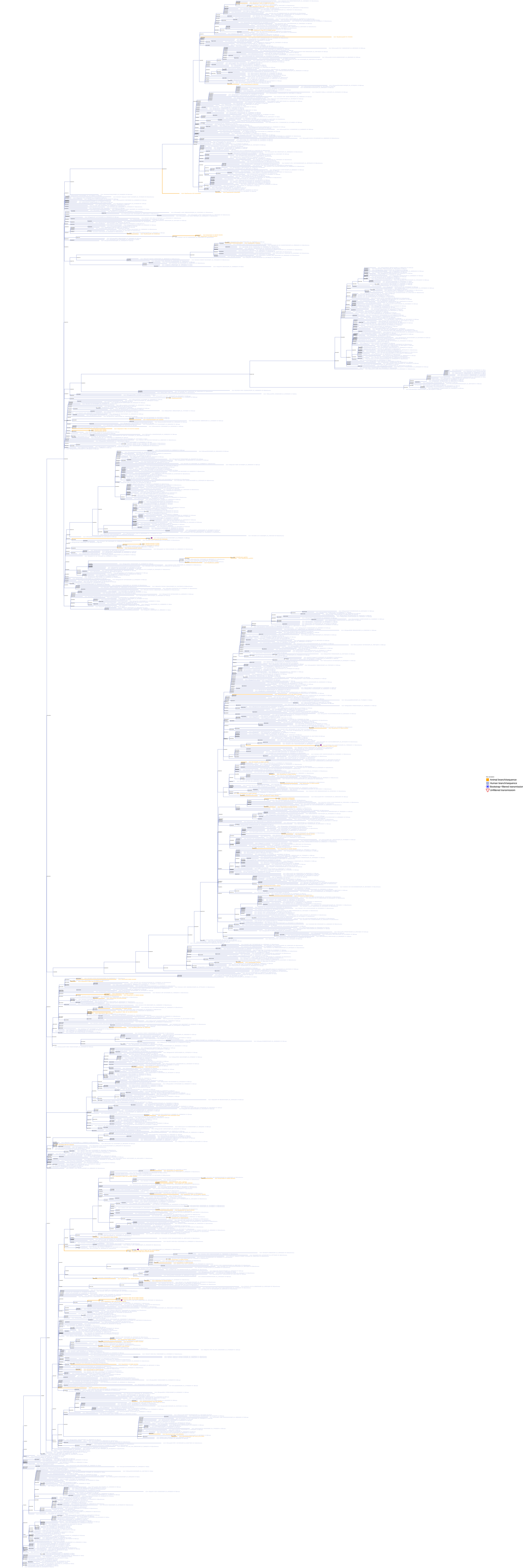

Tree annotation
Animal branch/sequence
Human branch/sequence
Bootstrap−filtered transmission
Unfiltered transmissiom
