# Supplementary material for: Zooanthroponotic transmission of SARS-CoV-2 and host-specific viral mutations revealed by genome-wide phylogenetic analysis
Source: eLife. 2023 Apr 4;12:e83685. doi: 10.7554/eLife.83685 (PMC10072876; doi:10.7554/eLife.83685)
Supplement: Figure 2—source data 4. — In order to make the tree topology clear, branch lengths are not to scale. The colored boxes to the right of the tree show the allelic state of the seven deer-associated genome-wide association studies (GWAS) hits that appeared in all ten replicate GWAS runs, with dark red indicating the animal-associated alternate allele. [file eLifeFigure2Figure Supplement 4.pdf › eLifeFigure2Figure Supplement 4.pdf]

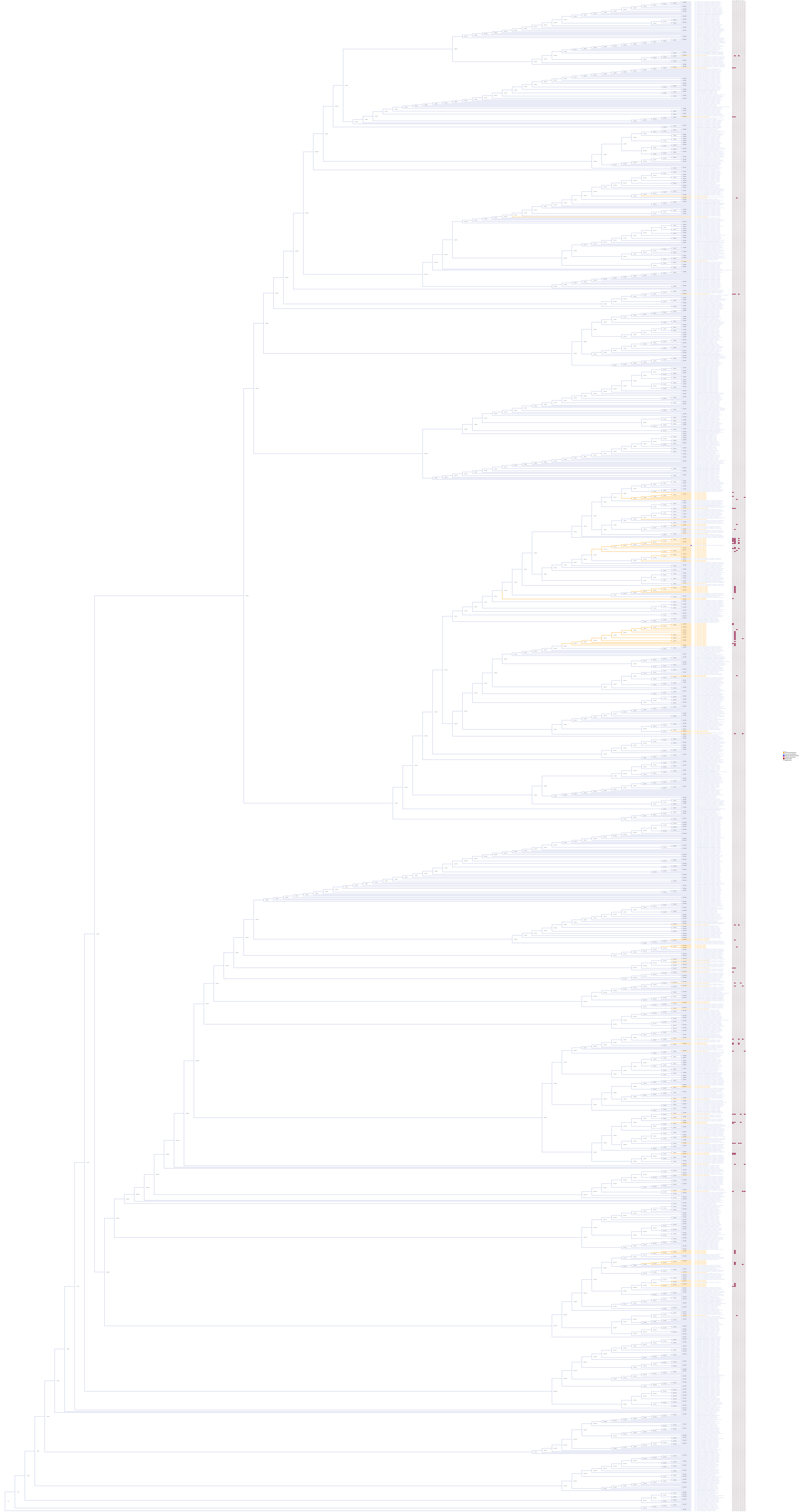
Animal branch/sequence
Human branch/sequence
Bootstrap-filtered transmission
Unfiltered transmission
Alternate Allele
Wildtype Allele
